# Supplementary material for: Frequency-encoded eye tracking smart contact lens for human–machine interaction
Source: Nat Commun. 2024 Apr 27;15:3588. doi: 10.1038/s41467-024-47851-y (PMC11055864; doi:10.1038/s41467-024-47851-y)
Supplement: Supplementary file 1 — Supplementary Information [file 41467_2024_47851_MOESM1_ESM.pdf]

Supplementary Information for

Frequency-encoded Eye Tracking Smart Contact Lens for Human-Machine Interaction

Authors: Hengtian Zhu[1†], Huan Yang[1†], Siqi Xu[2†], Yuanyuan Ma[3], Shugeng Zhu[1], Zhengyi Mao[1], Weiwei Chen[1,4,6], Zizhong Hu[2], Rongrong Pan[3], Yurui Xu[1,4,6], Yifeng Xiong[1], Ye Chen[5,1*], Yanqing Lu[1*], Xinghai Ning[1,4,6], Dechen Jiang[3,6], Songtao Yuan[2*], and Fei Xu[1,6*]

1. National Laboratory of Solid State Microstructures, College of Engineering and Applied Sciences, and Collaborative Innovation Center of Advanced Microstructures, Nanjing University, Nanjing 210023, China.
2. Department of Ophthalmology, The First Affiliated Hospital with Nanjing Medical University, Nanjing 210094, China.
3. The State Key Lab of Analytical Chemistry for Life Science, School of Chemistry and Chemical Engineering, Nanjing University, Nanjing 210093, China.
4. Jiangsu Key Laboratory of Artificial Functional Materials, Nanjing University, Nanjing 210093, China.
5. College of Physics, MIIT Key Laboratory of Aerospace Information Materials and Physics, State Key Laboratory of Mechanics and Control for Aerospace Structures, Nanjing University of Aeronautics and Astronautics, Nanjing 211106, China.
6. Chemistry and Biomedicine Innovation Center (ChemBIC), Nanjing University, Nanjing 210093, China.

† These authors contributed equally to this work.

* Email: yechen@nuaa.edu.cn; yqlu@nju.edu.cn; songtaoyuan@njmu.edu.cn; feixu@nju.edu.cn

This PDF file includes:

Supplementary Figures 1 to 35

Supplementary Tables 1 and 2

Supplementary Note 1

Other Supplementary Materials for this manuscript include the following:

Supplementary Videos 1 to 4

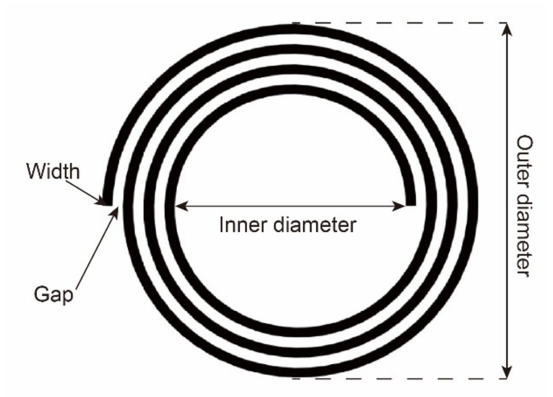

Supplementary Figure 1. Structural schematic illustration of the RF tag.

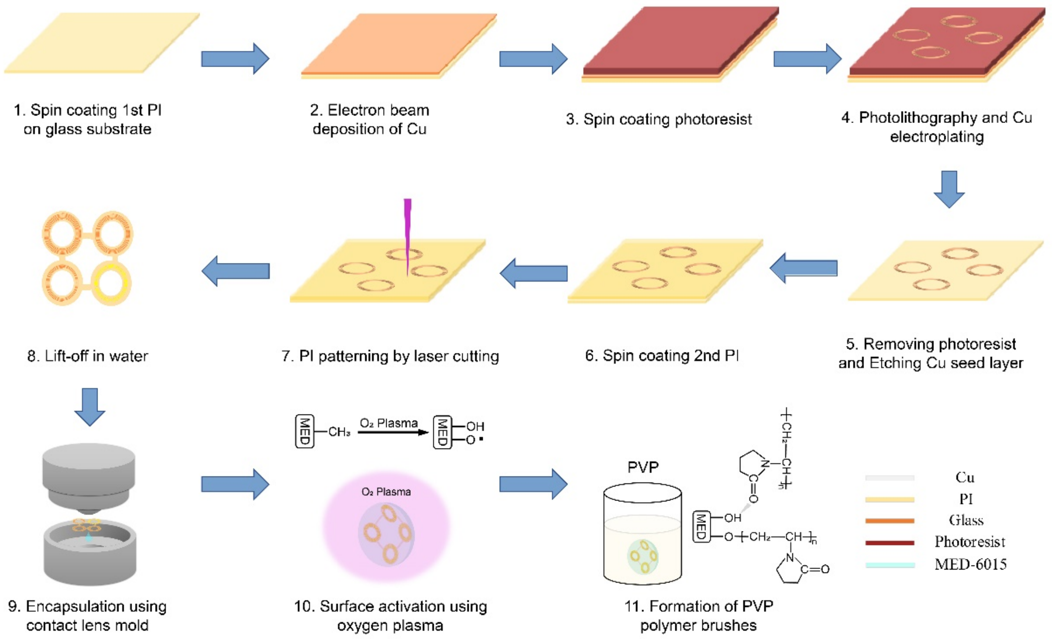

Supplementary Figure 2. Preparation process of SCL. Preparation process of SCL. The main process includes the micro/nano fabrication, laser engraving, precise encapsulation, and hydrophilic treatment.

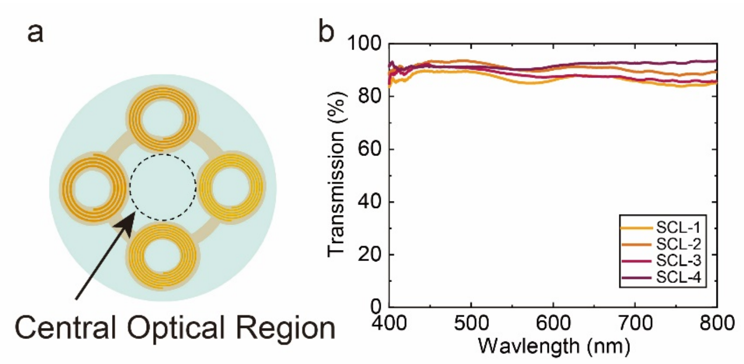

Supplementary Figure 3. a Schematic diagram of the central optical region of the SCL. **b** Transmission spectrum of the central optical region of the 4 SCLs.

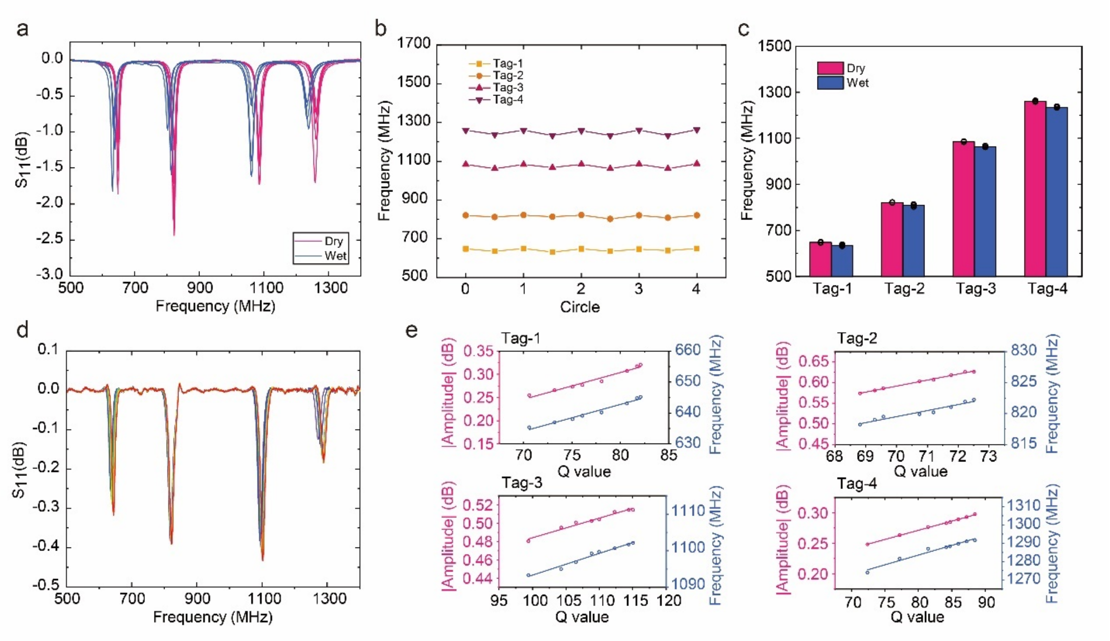

Supplementary Figure 4. Effect of water on the eye tracking SCL. S_{11} curves **a**, tags' frequency variation **b**, and frequency statistic **c** of the SCL under 4 circles of hydration and dehydration. In the frequency statistic, data are presented as mean with standard deviation of 4 circles of hydration and dehydration. **d** S_{11} curves of the SCL during the dehydration process. **e** Amplitude and frequency increased with the Q value for the 4 tags.

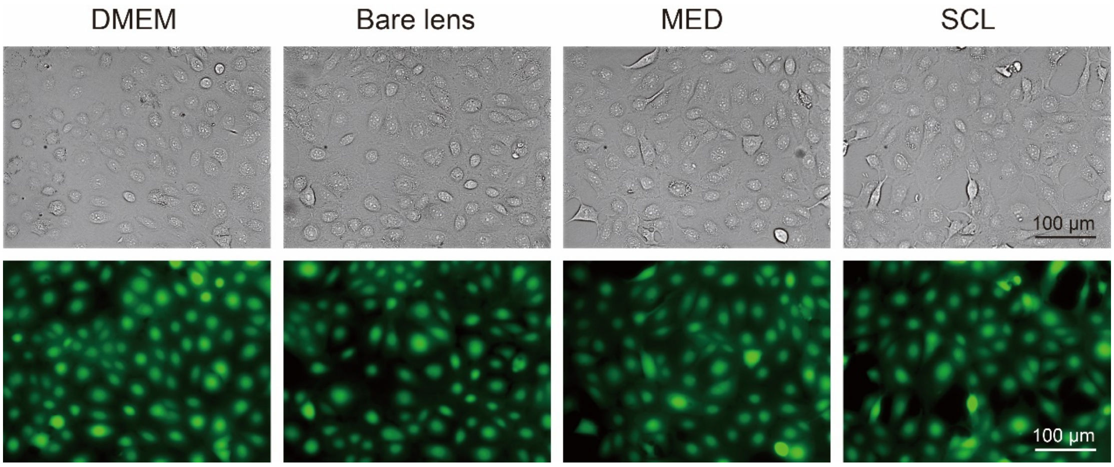

Supplementary Figure 5. Micrographs and fluorescence images of HCE-T incubated in different extracts. The cell distribution density and single cell's fluorescence intensity of HCE-T incubated in different extracts were similar, indicating low bio-toxicity.

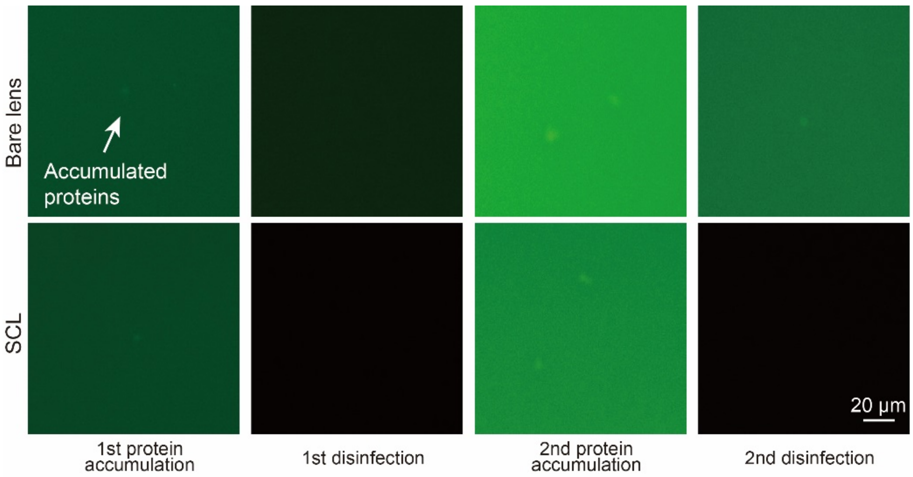

Supplementary Figure 6. Representative fluorescence image of the bare commercial contact lens (top row) and the SCL (bottom row) after biperiodic protein accumulation and disinfection.

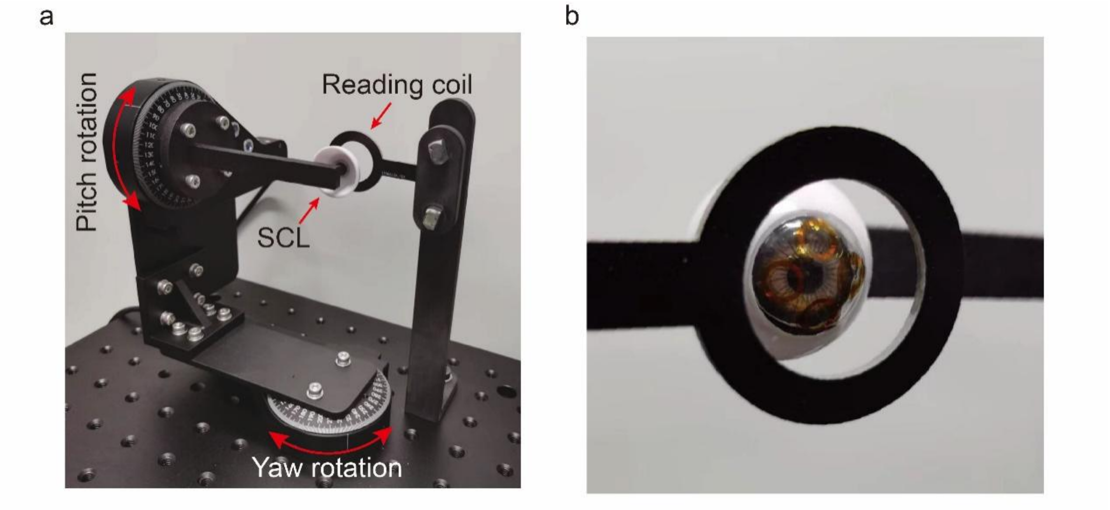

Supplementary Figure 7. Photographs of 2D eye movement model. a Eye movement model composed of 2 rotating platforms that actuate pitch rotation and yaw rotation of the SCL respectively and one reading coil placed in front of the SCL. **b** Details of the SCL and reading coil.

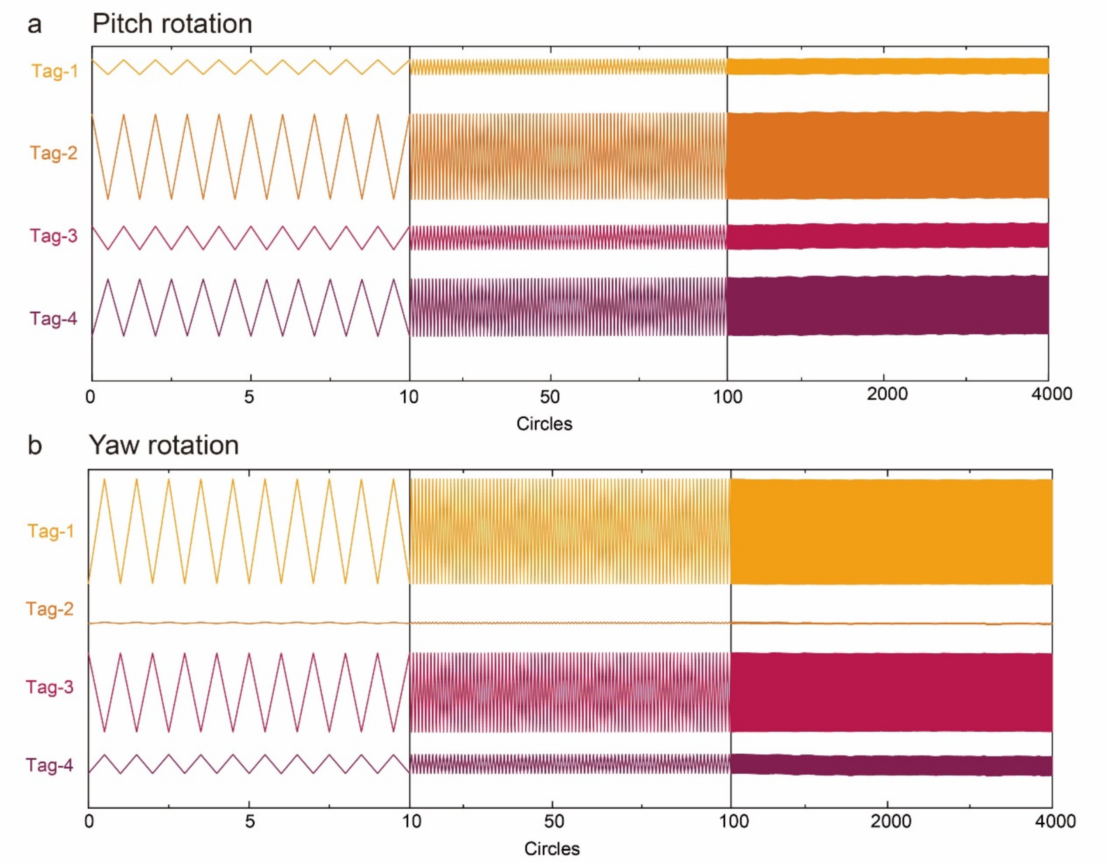

Supplementary Figure 8. Repeatability test of SCL. Responses of eye movement with pitch angles of ±10° **a** and yaw angles of ±10° **b**. Repeat times: 4000.

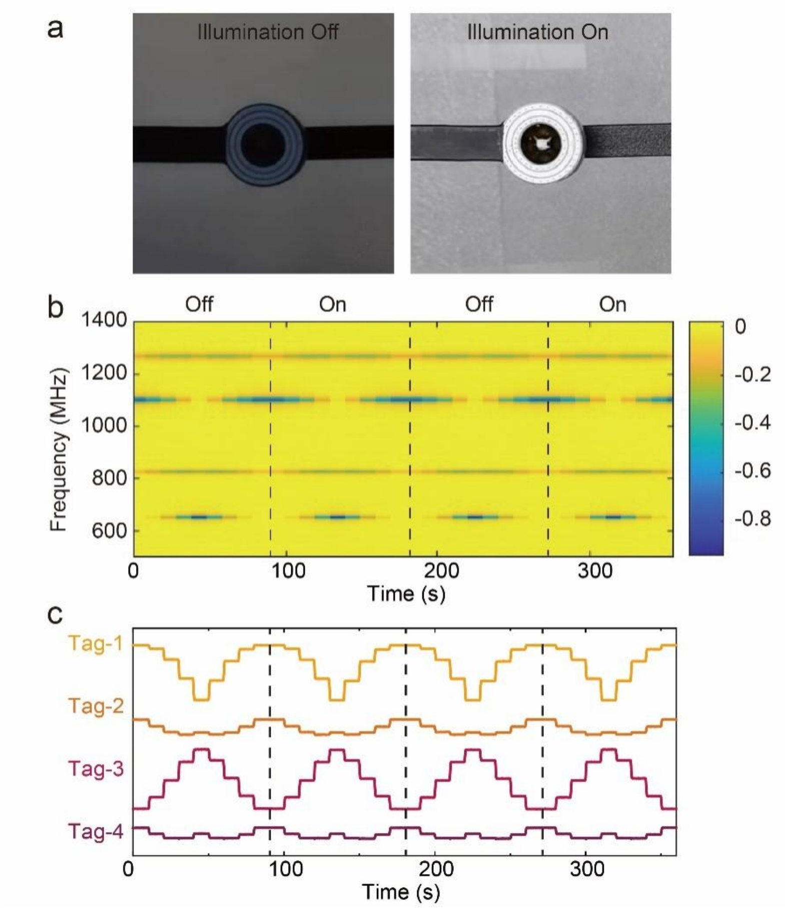

Supplementary Figure 9. Anti-illumination test. a Photographs of the SCL when the illumination was off and on. Dynamic S_{11} curve **b** and tags' responses **c** of the SCL under different yaw angles when the illumination was off and on.

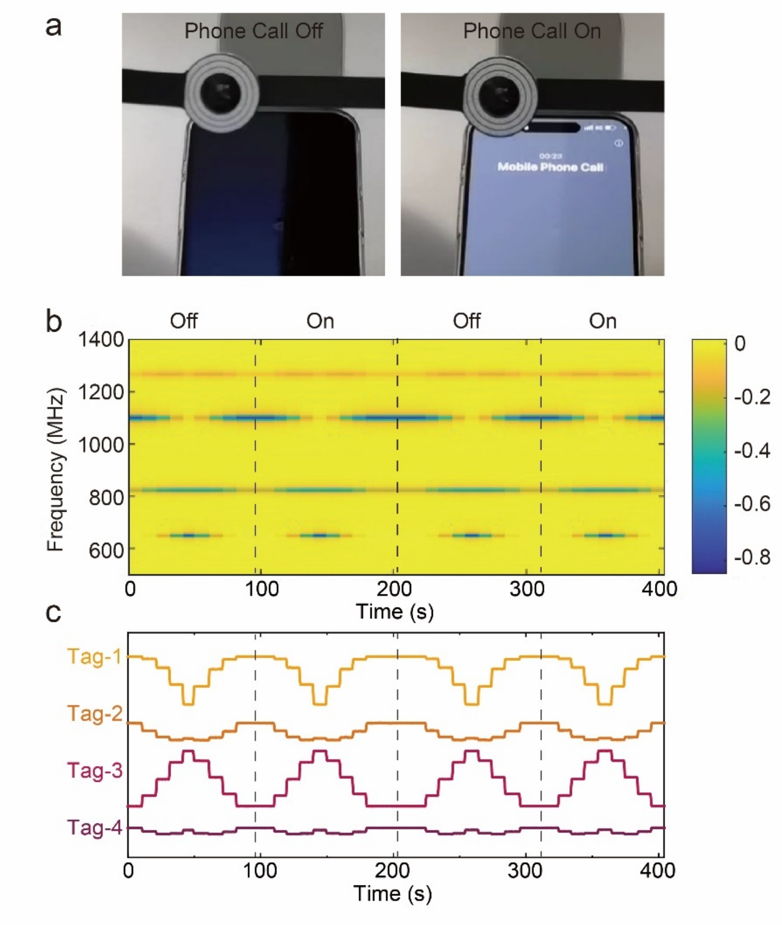

Supplementary Figure 10. RF immunity test (from smartphones). a Photographs of the SCL when the mobile phone call was off and on. Dynamic S_{11} curve **b** and tags' responses **c** of the SCL under different yaw angles when the mobile phone call was off and on.

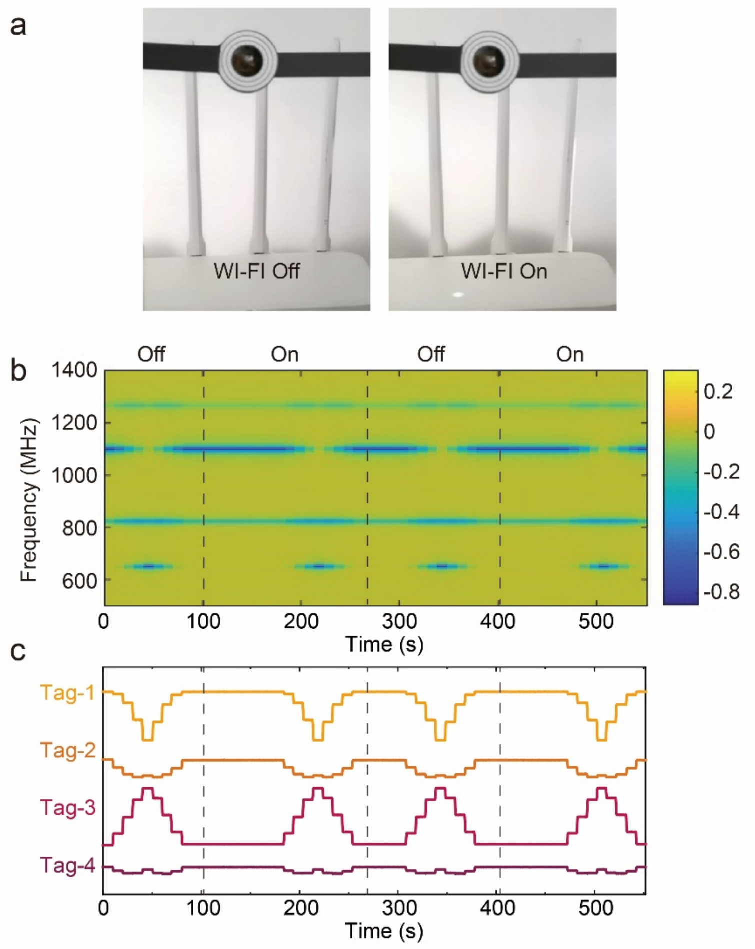

Supplementary Figure 11. RF immunity test (from Wi-Fi router). a Photographs of the SCL when the Wi-Fi router was off and on. Dynamic S_{11} curve **b** and tags' responses **c** of the SCL under different yaw angles when the Wi-Fi router was off and on.

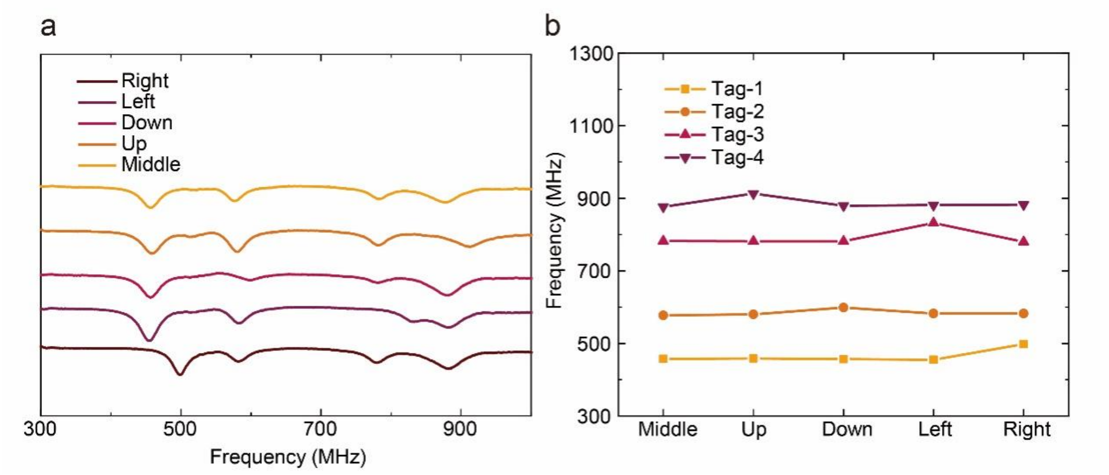

Supplementary Figure 12. Slippage test of the SCL. S_{11} curve **a** and tags' frequency variation **b** of the SCL at the center and surrounding location of the cornea.

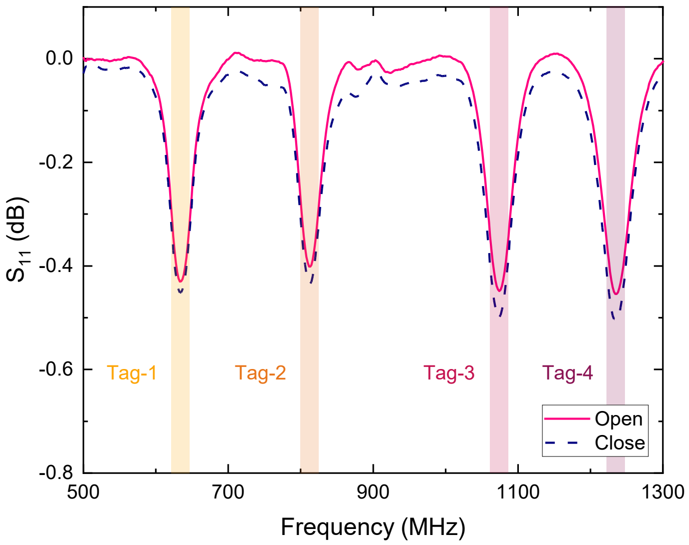

Supplementary Figure 13. S_{11} curves of SCL when eye opened and closed.

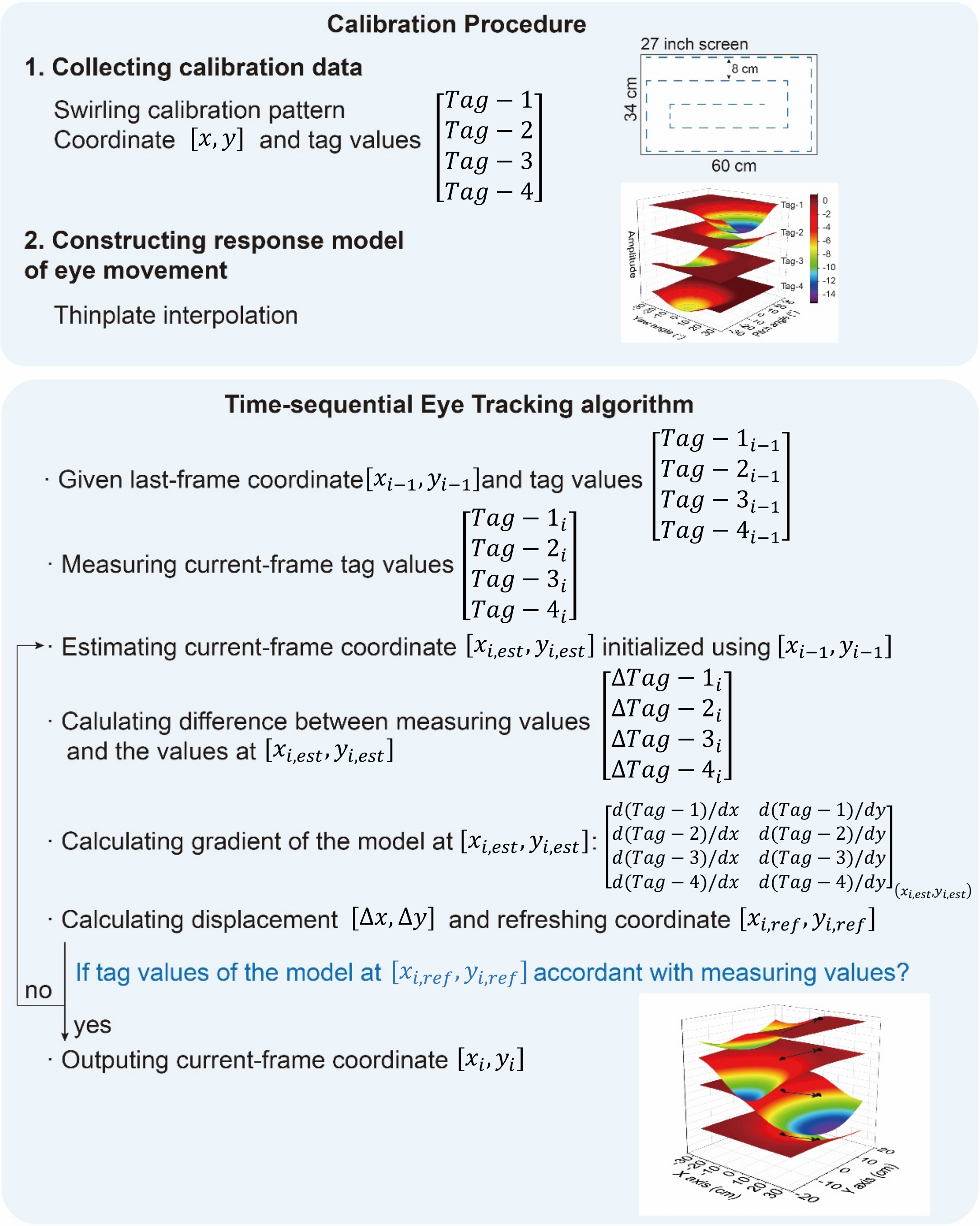

Supplementary Figure 14. Summary of the calibration procedure and time-sequential eye tracking algorithm.

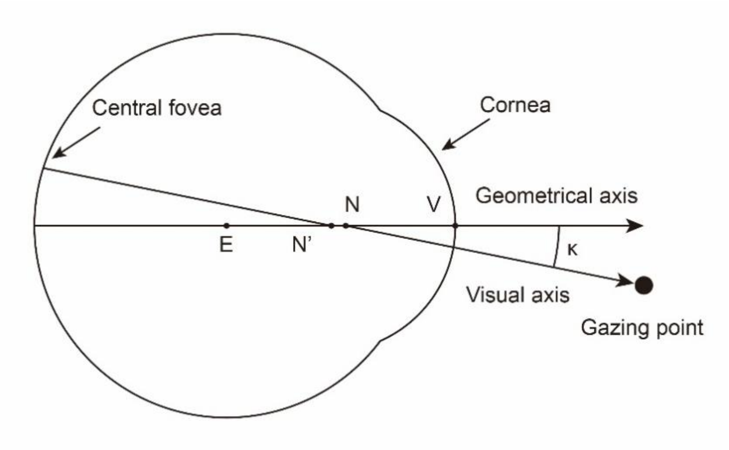

Supplementary Figure 15. Schematic diagram of anatomy and optical path of eye.

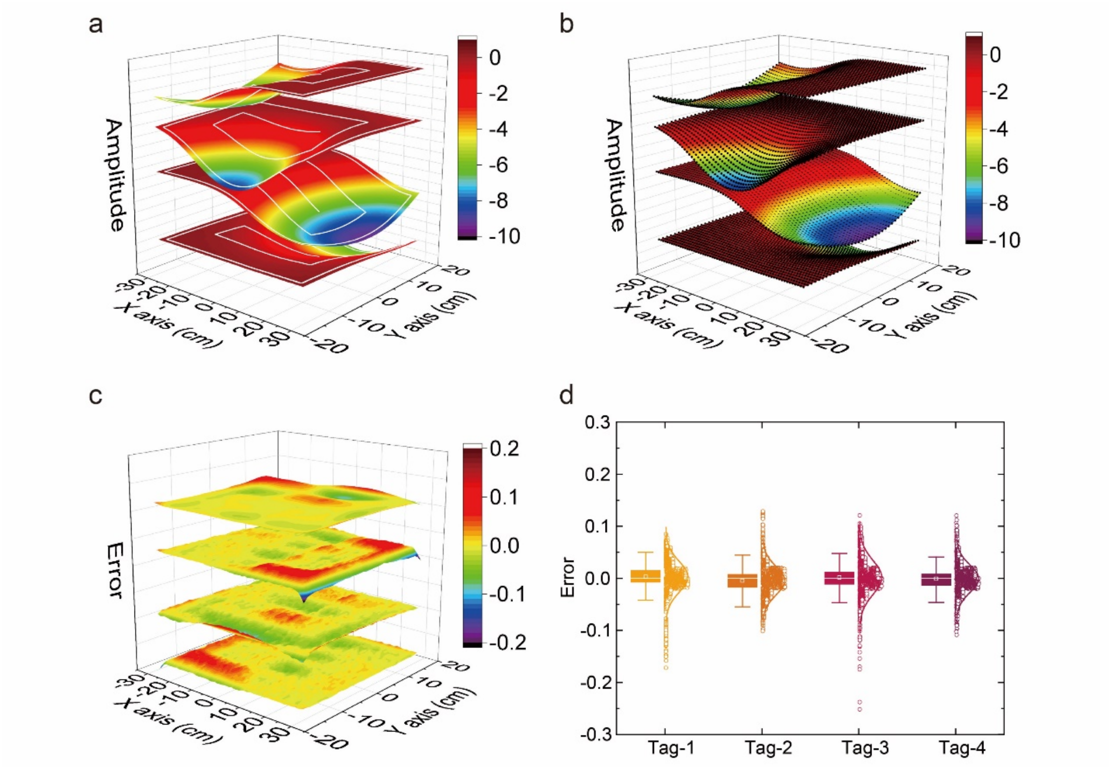

Supplementary Figure 16. Accuracy of eye-movement model using implicit swirling calibration method. a Constructed eye-movement model using implicit swirling calibration method. The white traces indicate swirling calibration data. **b** Comparison between swirling-calibration model and fingerprint model. The black dots indicate the fingerprint data collected by traversing over the entire screen region. **c** Spatially distributed error of swirling-calibration model compared with fingerprint data. **d** Error statistics of multiple tags. The middle line is determined by median, the box is determined by the 25th and 75th percentiles, and the whiskers are determined by the 5th and 95th percentiles. n = 2135 independent gazing points.

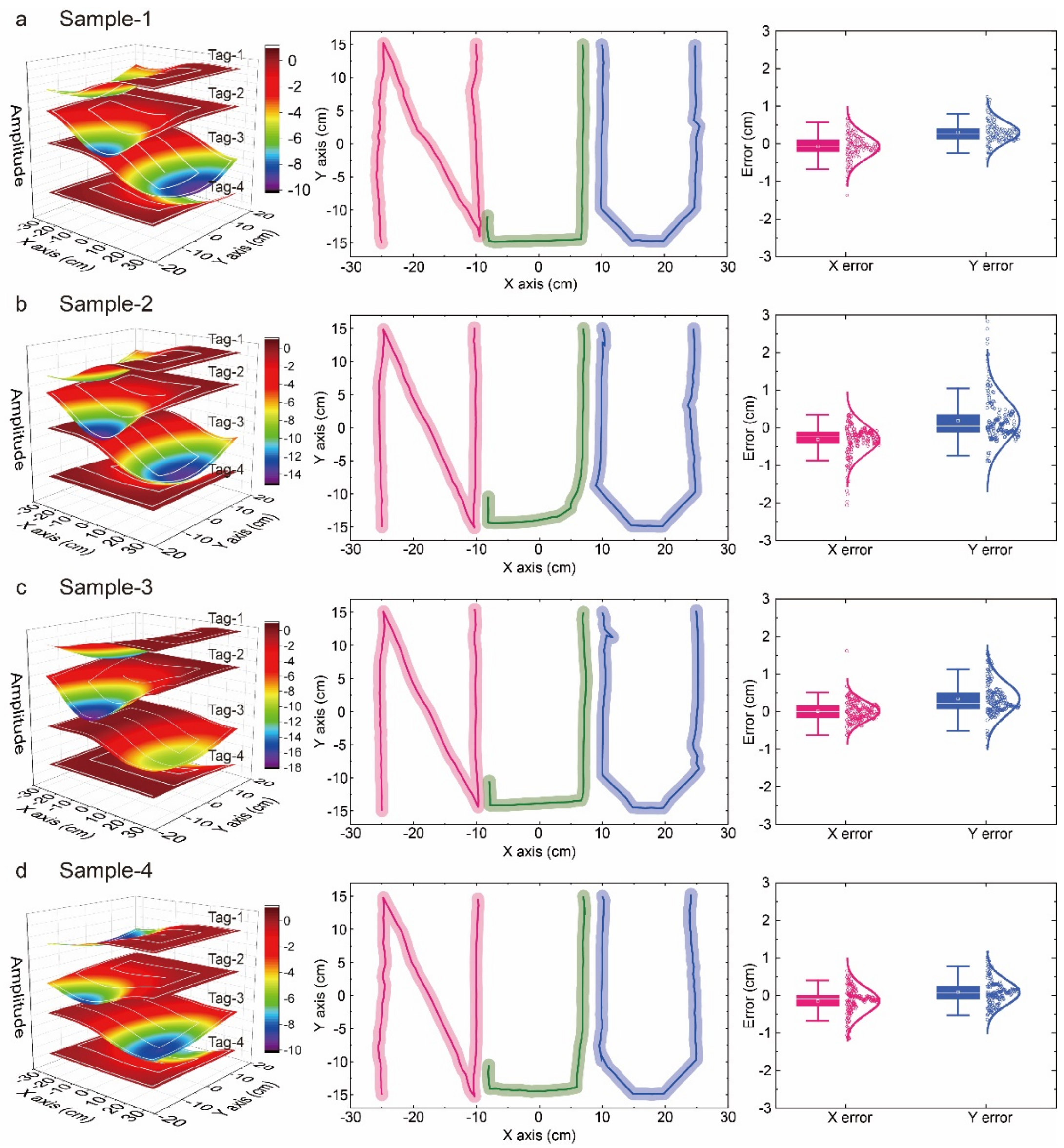

Supplementary Figure 17. Response models, eye-drawing NJU letters, and error statistic of 4 samples. In the error statistic, the middle line is determined by median, the box is determined by the 25th and 75th percentiles, and the whiskers are determined by the 5th and 95th percentiles. n = 204 independent gazing points.

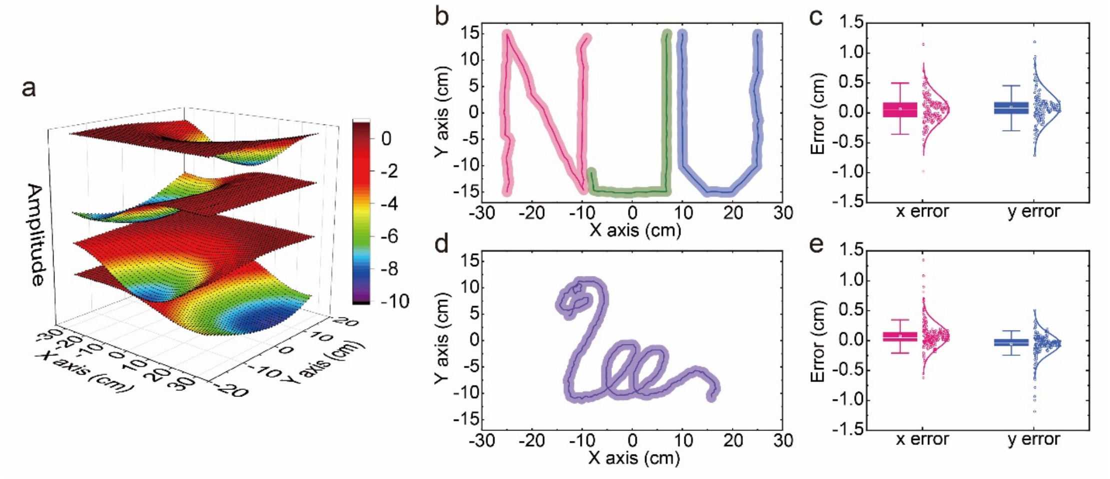

Supplementary Figure 18. Calligraphy and painting using fingerprint model. a Constructed eye-movement fingerprint model by traversing the signal over the entire screen region. **b-e** Eye-drawing NJU letters and snake pattern with low horizontal and vertical error. Width of semitransparent trace indicates fixation range of central fovea. In the error statistic, the middle line is determined by median, the box is determined by the 25th and 75th percentiles, and the whiskers are determined by the 5th and 95th percentiles. *n* = 204 independent gazing points for NJU letters, and *n* = 349 independent gazing points for snake pattern.

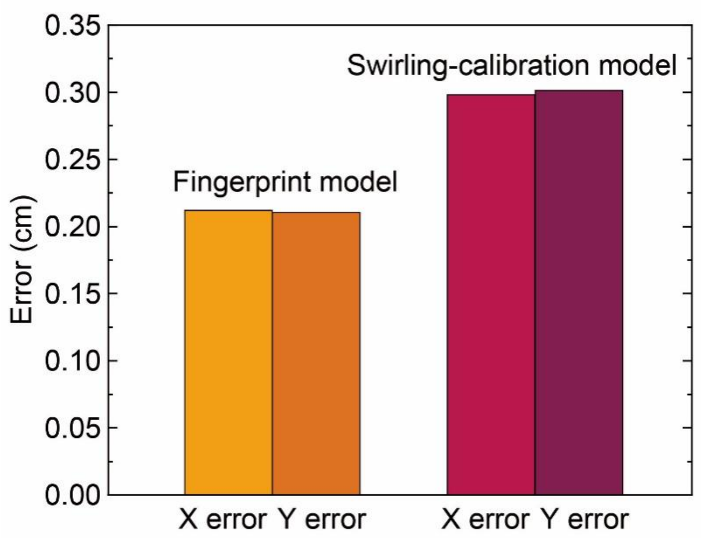

Supplementary Figure 19. Errors of eye-drawing patterns using fingerprint model and swirling-calibration model. The error using fingerprint model was mostly caused by the signal noise from the VNA. More error using swirling-calibration resulted from less accuracy of the constructed response model.

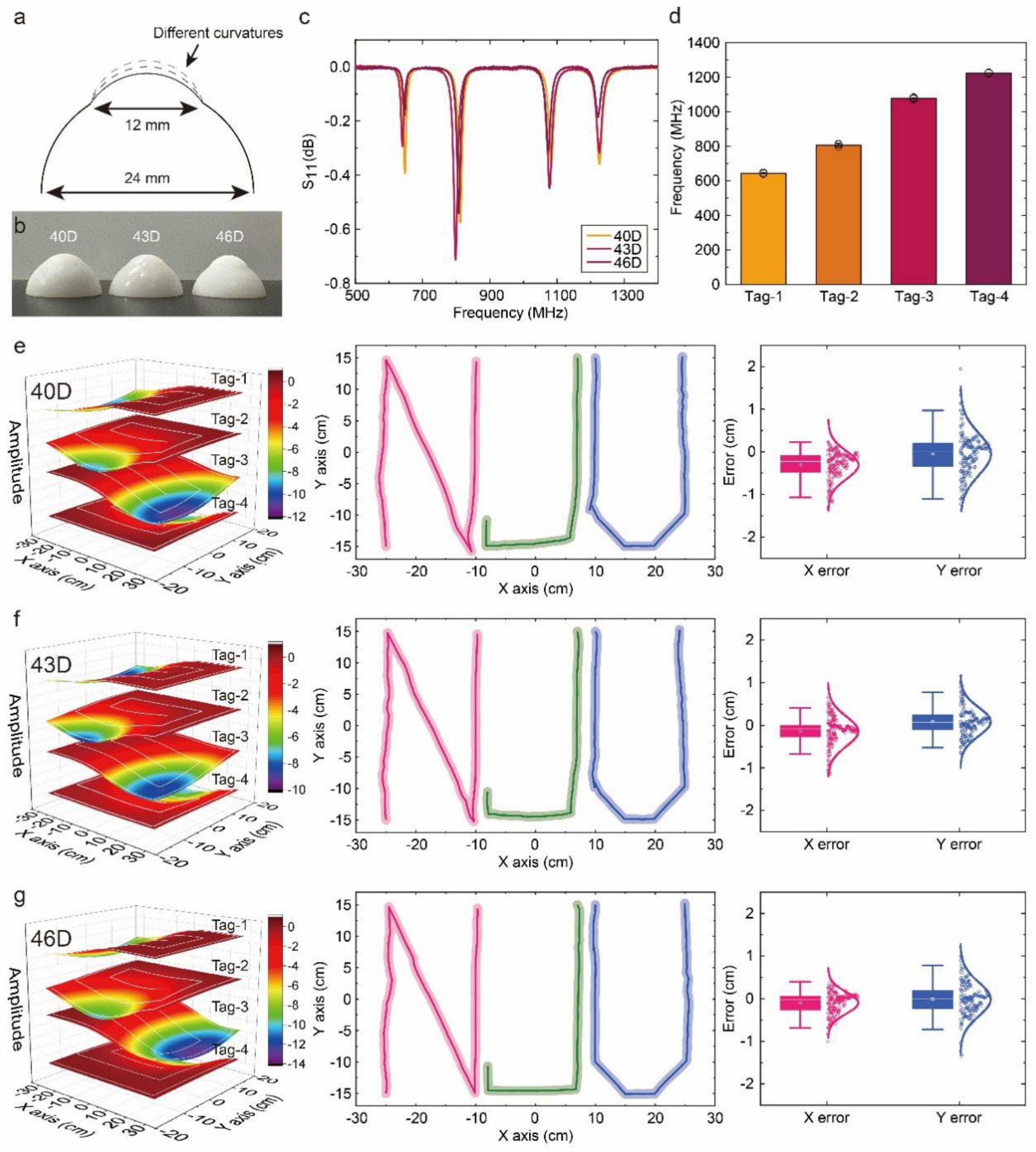

Supplementary Figure 20. Characterization of eye track SCL worn on corneas of different curvatures. a Structural schematic diagram of eyeball model with different curvatures. **b** Photographs of 3 eyeball models with corneal curvatures of 40D, 43D, and 46D. **c** S_{11} curves of SCL worn on the eyeball models. **d** Statistic of the 4 tags' working frequency. Data are presented as mean with standard deviation of multiple tests: n = 3. **e-g** Response models, eye-drawing NJU letters, and error analysis of SCL worn on the eyeball models with 3 different curvatures. In the error statistic, the middle line is determined by median, the box is determined by the 25th and 75th percentiles, and the whiskers are determined by the 5th and 95th percentiles. *n* = 204 independent gazing points.

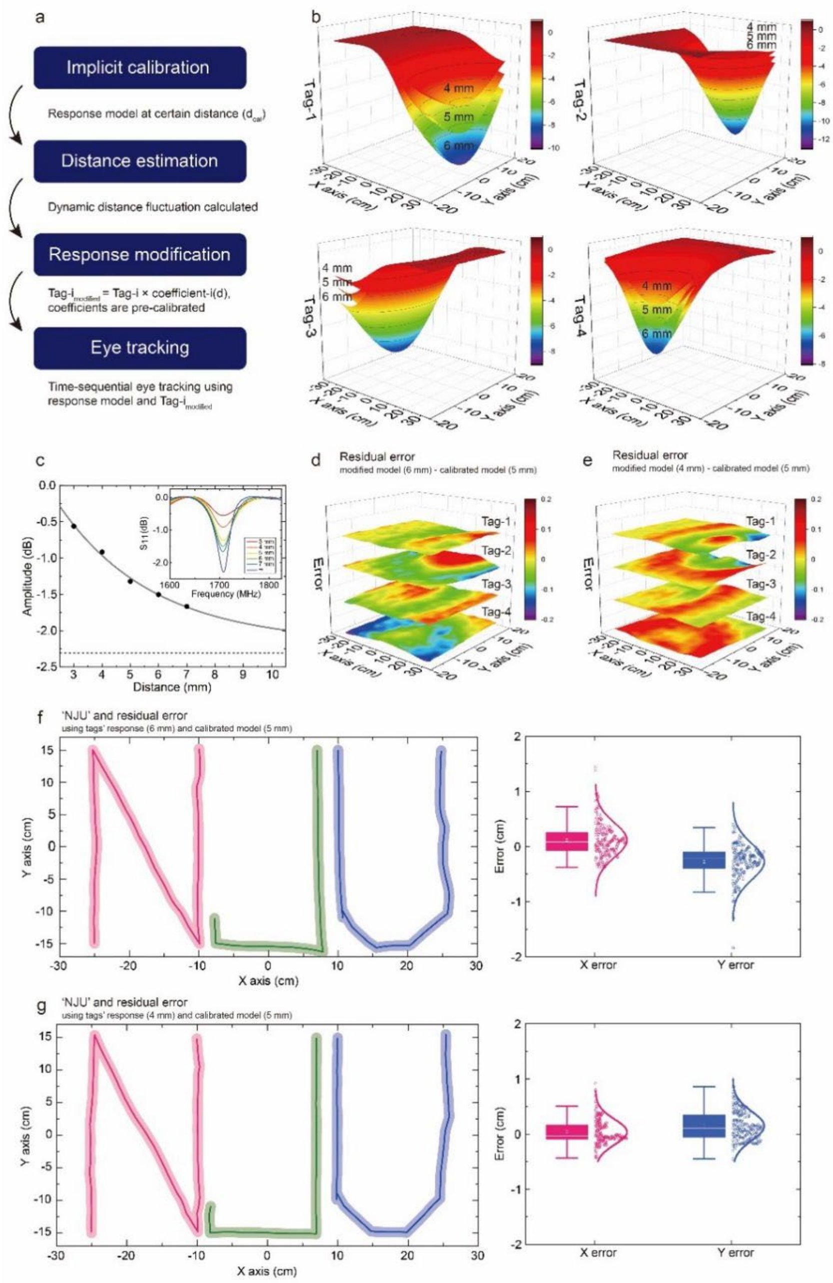

Supplementary Figure 21. Optimized eye tracking algorithm for resistance to reading distance variation. **a** Procedure of the optimized eye tracking algorithm. **b** Response models of 4 RF tags under different reading distances between the receiver coil and the SCL. **c** Amplitude of the self-resonant signal of the receiver coil with different distances away from a porcine eye. Inset: S_{11} curves of the receiver coil. Residual error of the modified response detected at a distance of 6 mm **d** and 4 mm **e**, compared to the response model calibrated at the distance of 5 mm. Eye-drawing NJU letters with low horizontal and vertical error using tags' response detected at a distance of 6 mm **f** and 4 mm **g** based on the calibrated model (5 mm). In the error statistic, the middle line is determined by median, the box is determined by the 25th and 75th percentiles, and the whiskers are determined by the 5th and 95th percentiles. *n* = 204 independent gazing points.

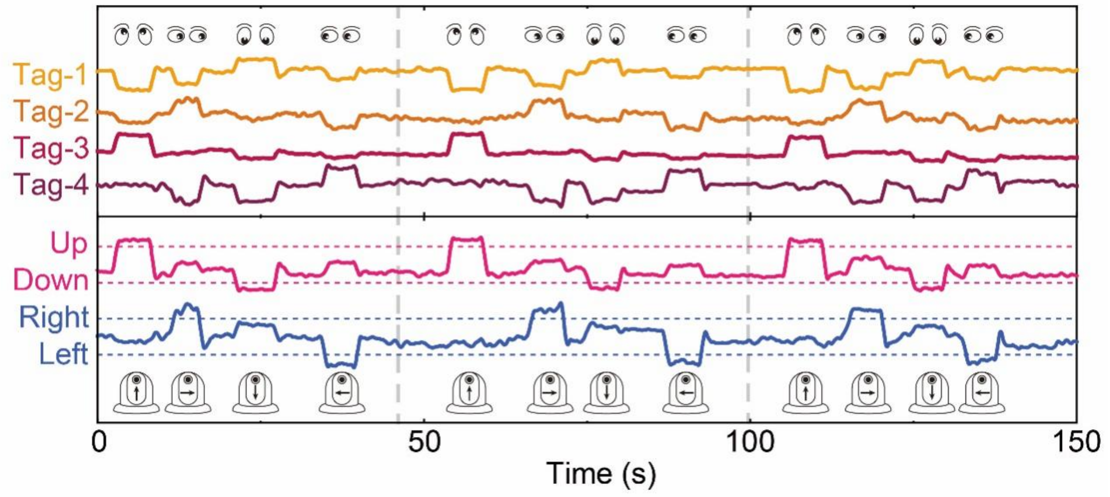

Supplementary Figure 22. Raw signals and eye expression demodulation when interacting with PTZ camera. 3-turns eye expression (up, right, down, and left) is applied to handle the PTZ camera.

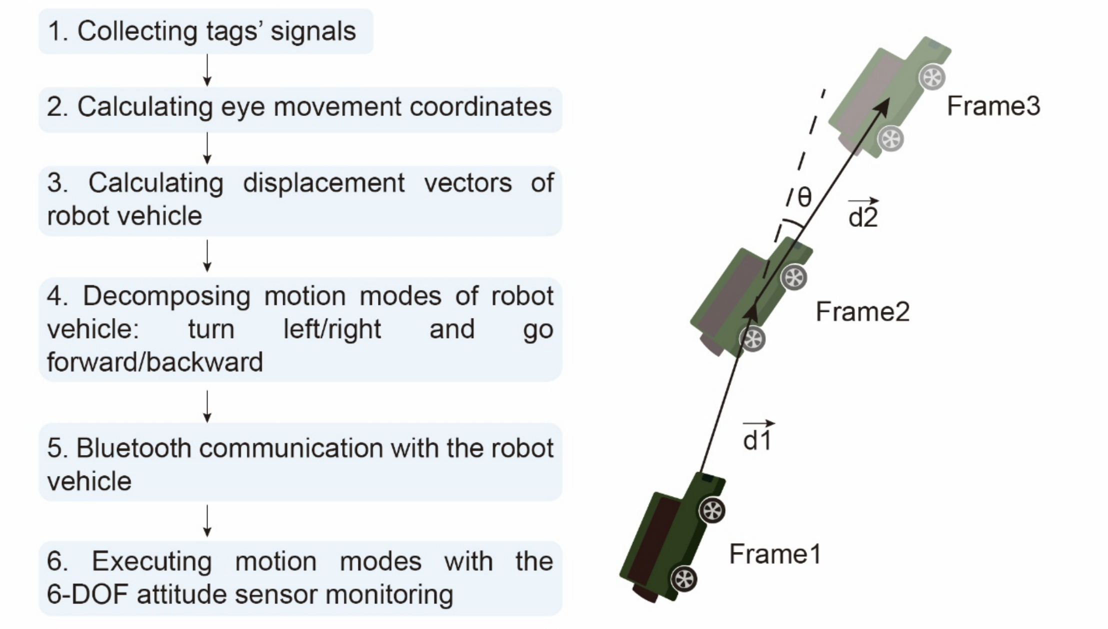

Supplementary Figure 23. Procedure and Schematic illustration of eye-controlled robot vehicle.

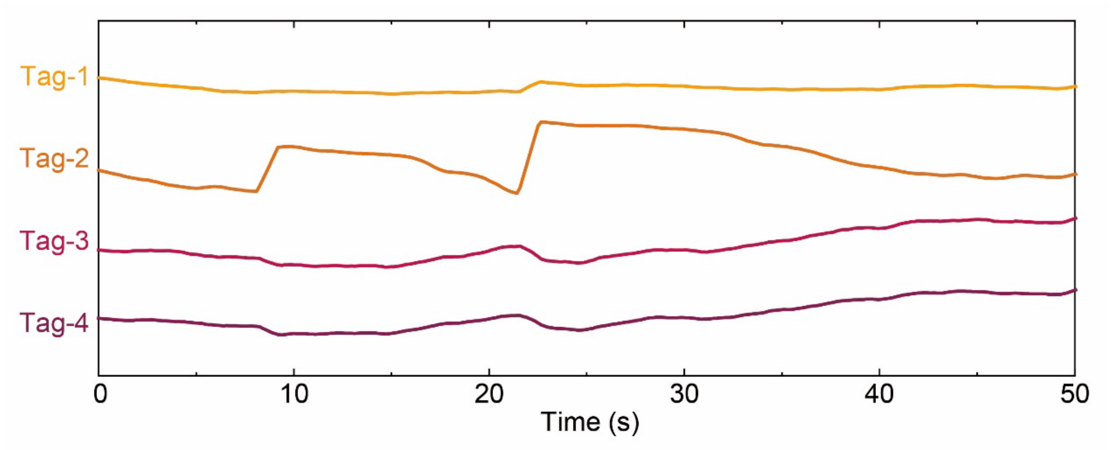

Supplementary Figure 24. Raw signals of SCL when rabbit eye controlled the robot vehicle.

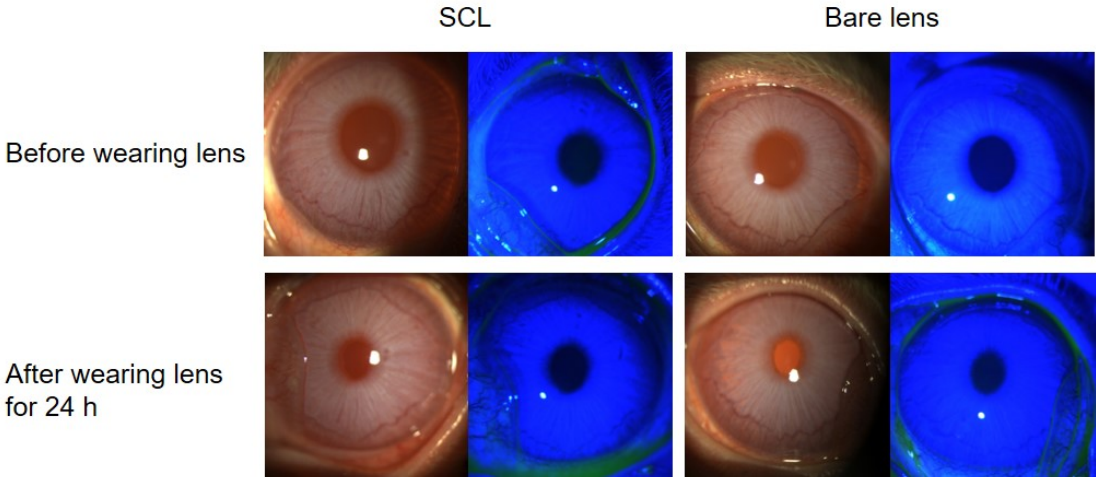

Supplementary Figure 25. Slit lamp examination using rabbits. No corneal injury was observed both in slit lamp micrographs and fluorescent images after 24-h wear of SCL and bare lens, indicating low eye irritation.

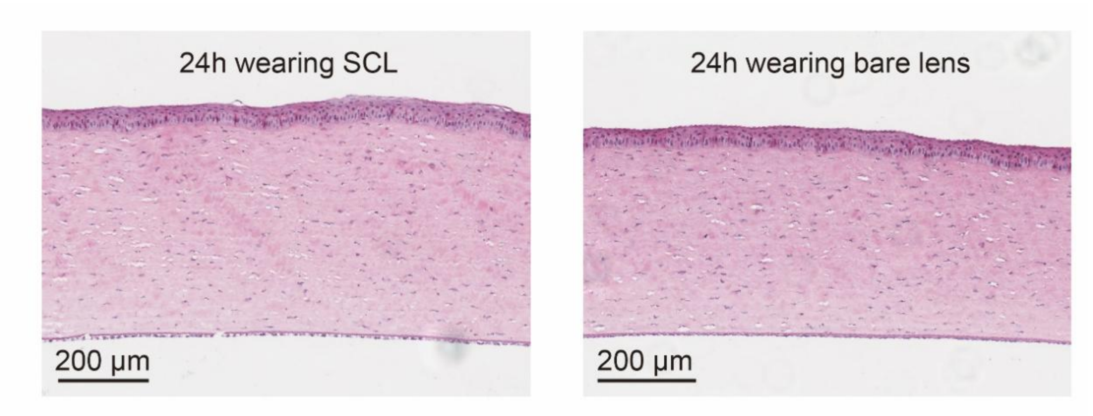

Supplementary Figure 26. Micrographs of the corneal tissue of the rabbit eyes with H&E staining after 24-h wear of the SCL and bare lens.

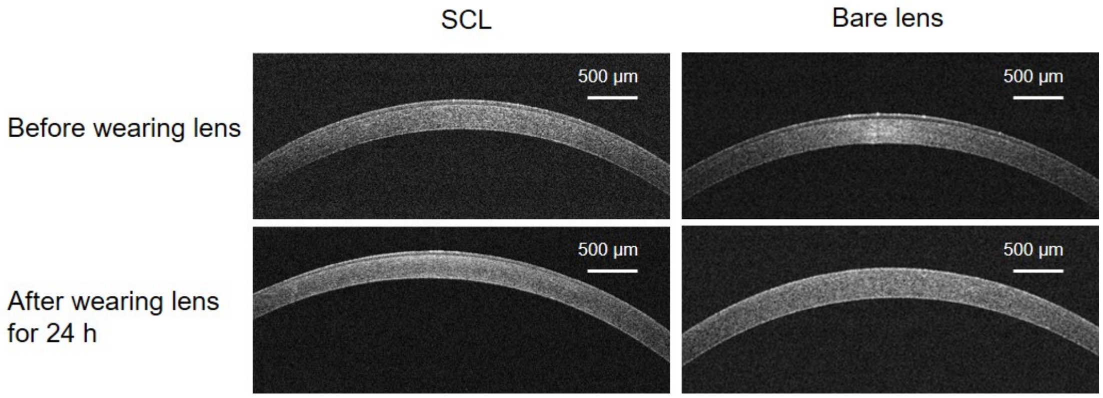

Supplementary Figure 27. OCT images of rabbit eye before and after 24-h wear of SCL and bare lens. No corneal injury or edema was observed in the rabbit eyes, indicating low eye irritation of SCL.

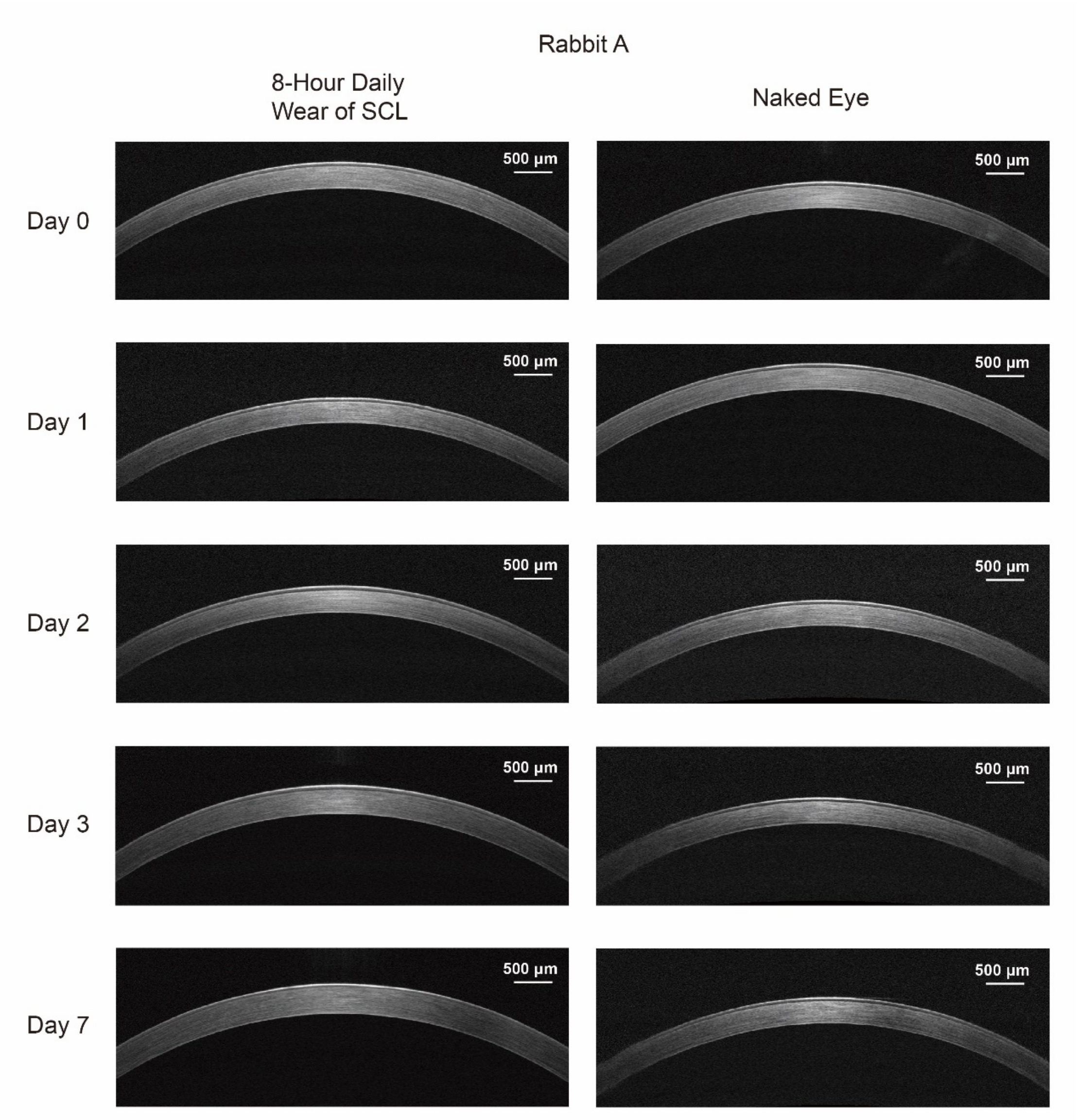

Supplementary Figure 28. OCT images of Rabbit A's eye after wearing the SCL for 8 hours daily over 1 week (left column), in comparison to the naked eye (right column).

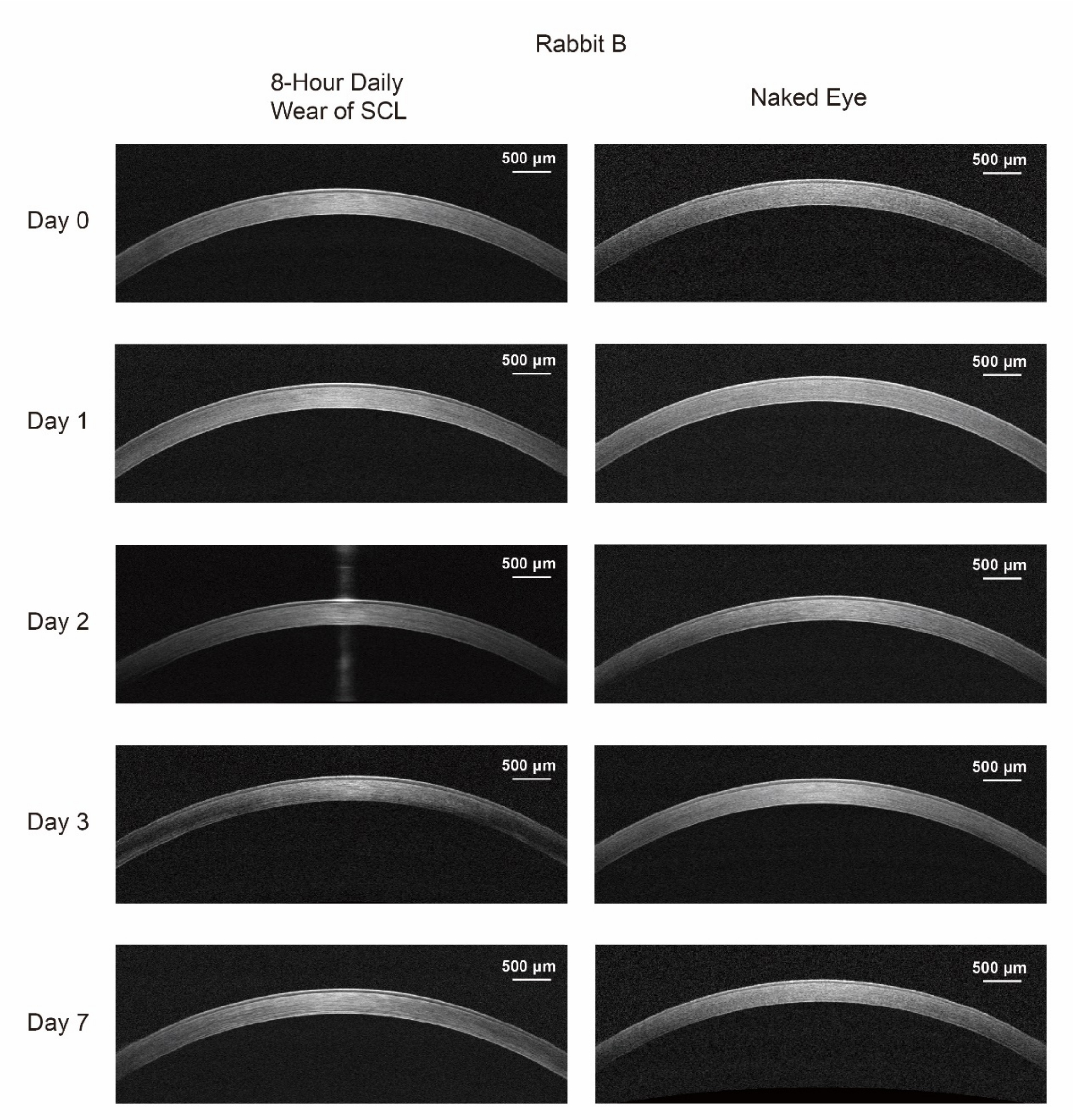

Supplementary Figure 29. OCT images of Rabbit B's eye after wearing the SCL for 8 hours daily over 1 week (left column), in comparison to the naked eye (right column).

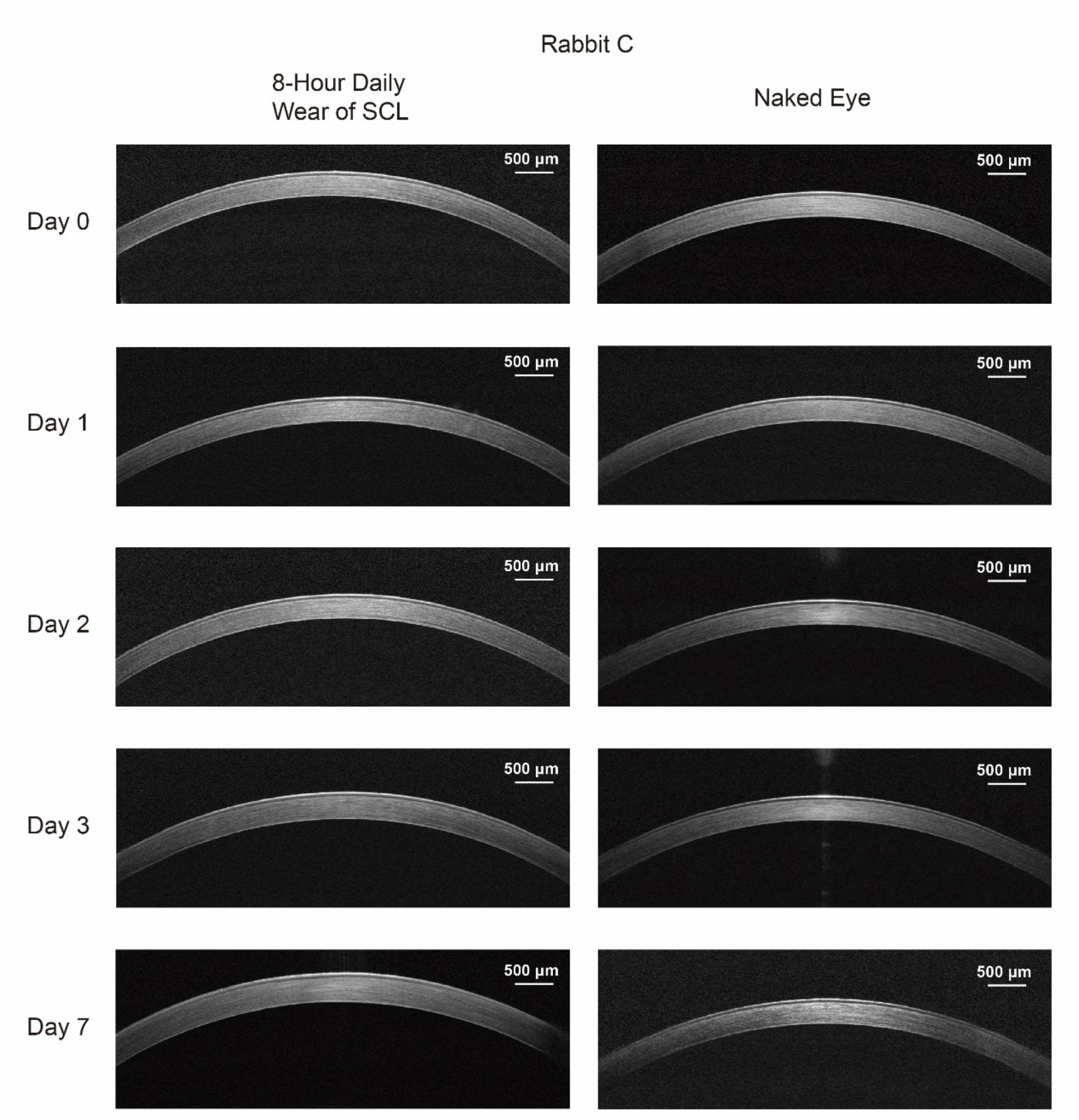

Supplementary Figure 30. OCT images of Rabbit C's eye after wearing the SCL for 8 hours daily over 1 week (left column), in comparison to the naked eye (right column).

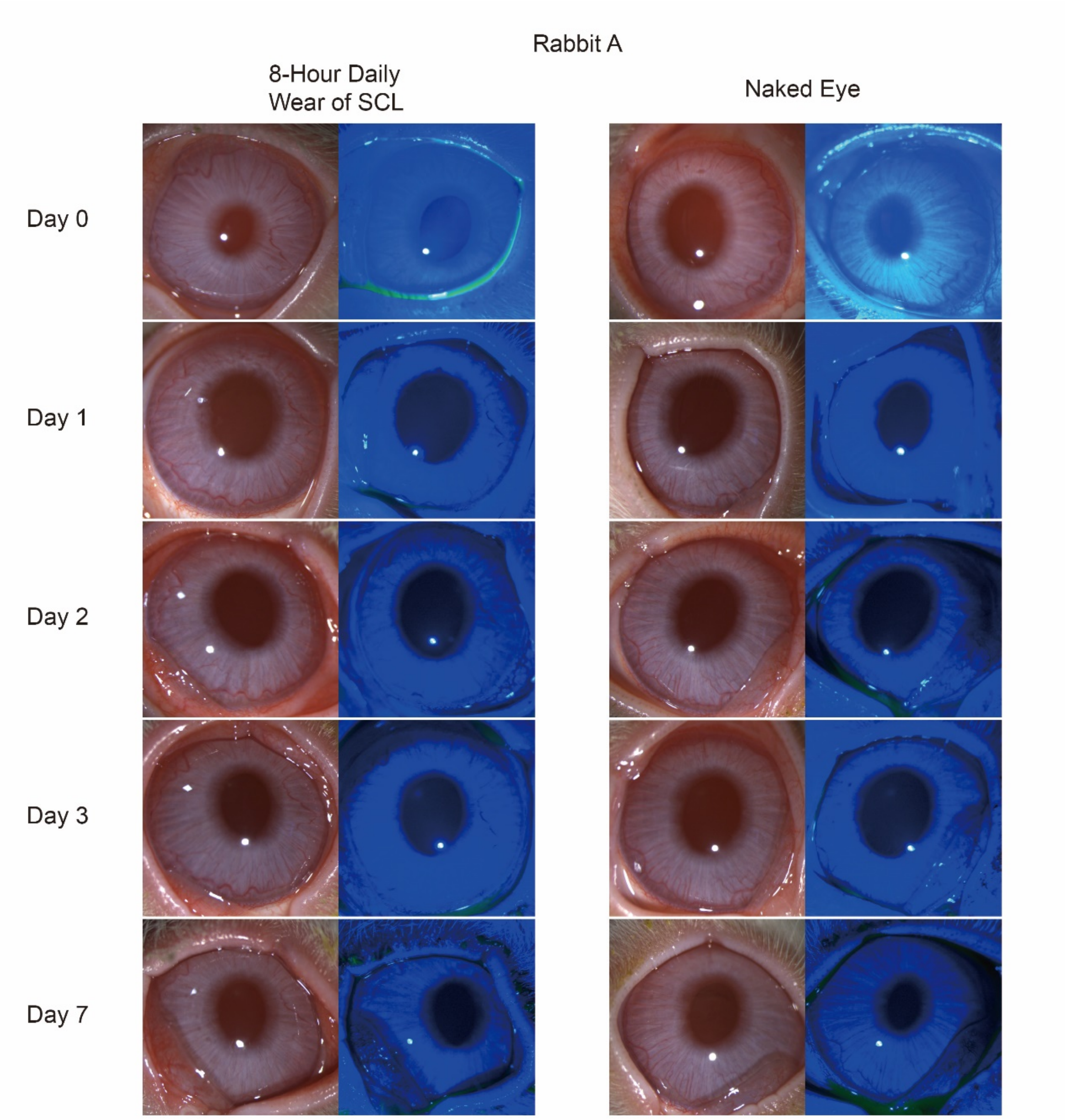

Supplementary Figure 31. Slit lamp micrographs and fluorescent images of Rabbit A's eye after wearing the SCL for 8 hours daily over 1 week (left column), in comparison to the naked eye (right column).

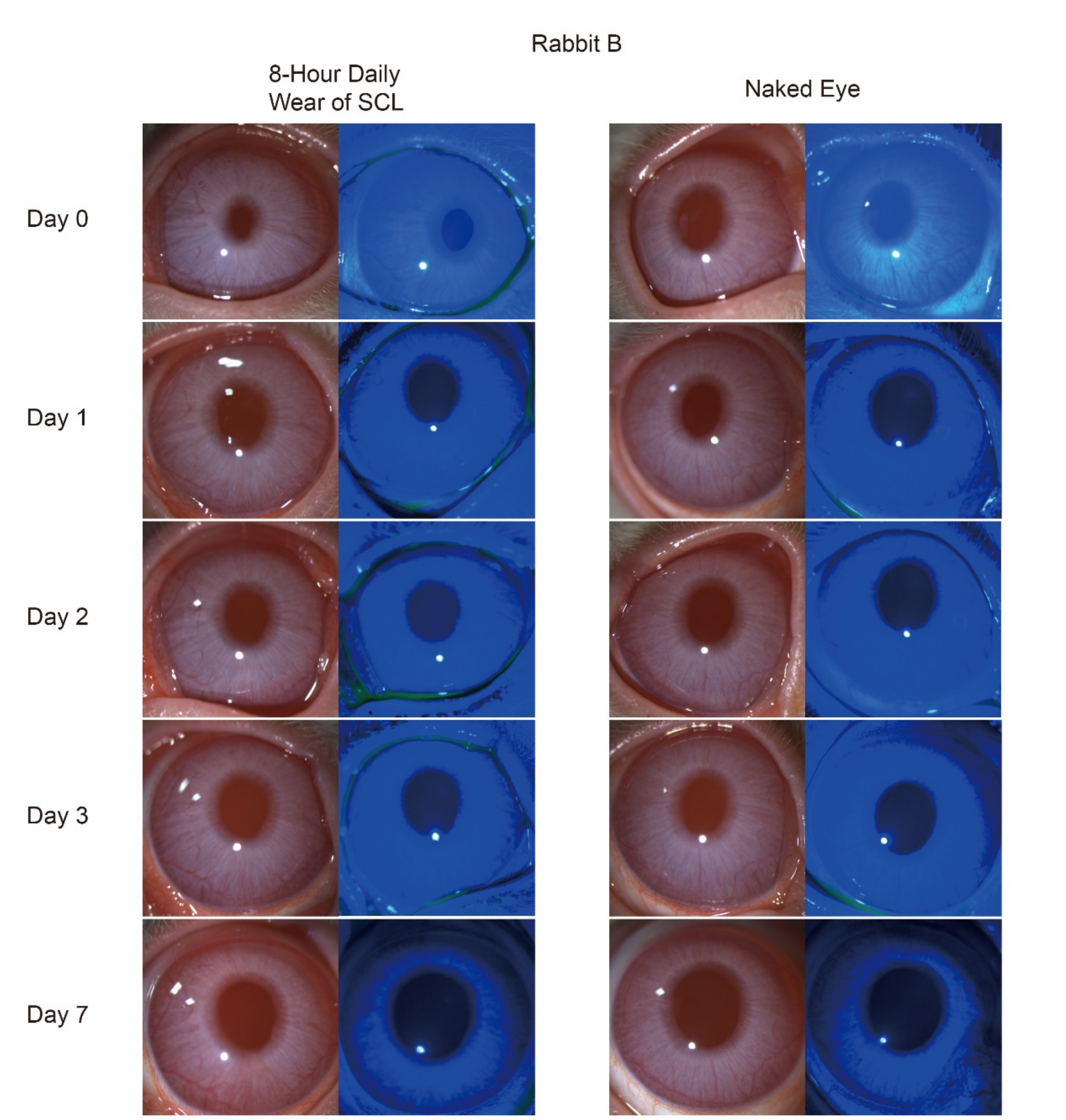

Supplementary Figure 32. Slit lamp micrographs and fluorescent images of Rabbit B's eye after wearing the SCL for 8 hours daily over 1 week (left column), in comparison to the naked eye (right column).

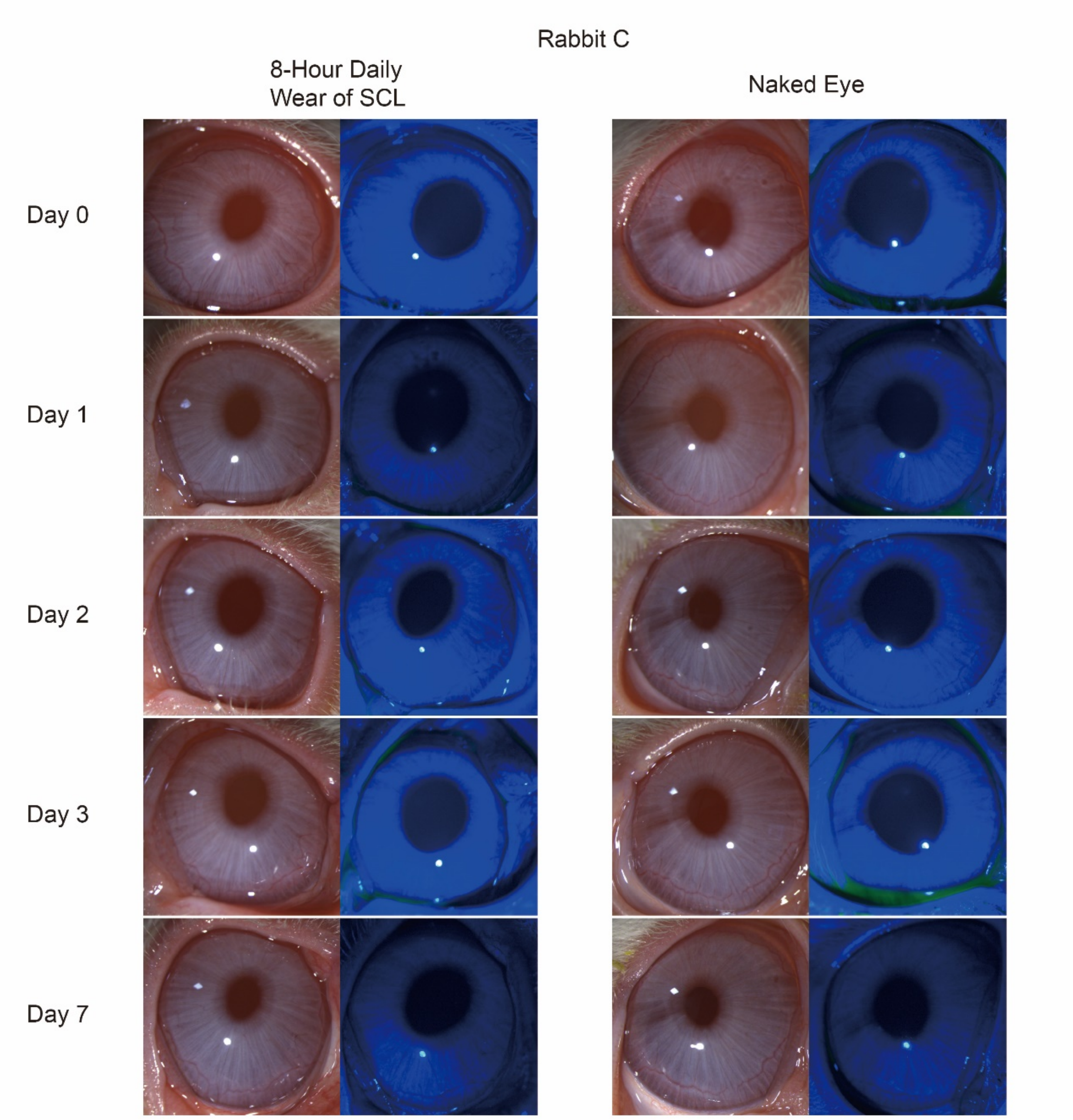

Supplementary Figure 33. Slit lamp micrographs and fluorescent images of Rabbit C's eye after wearing the SCL for 8 hours daily over 1 week (left column), in comparison to the naked eye (right column).

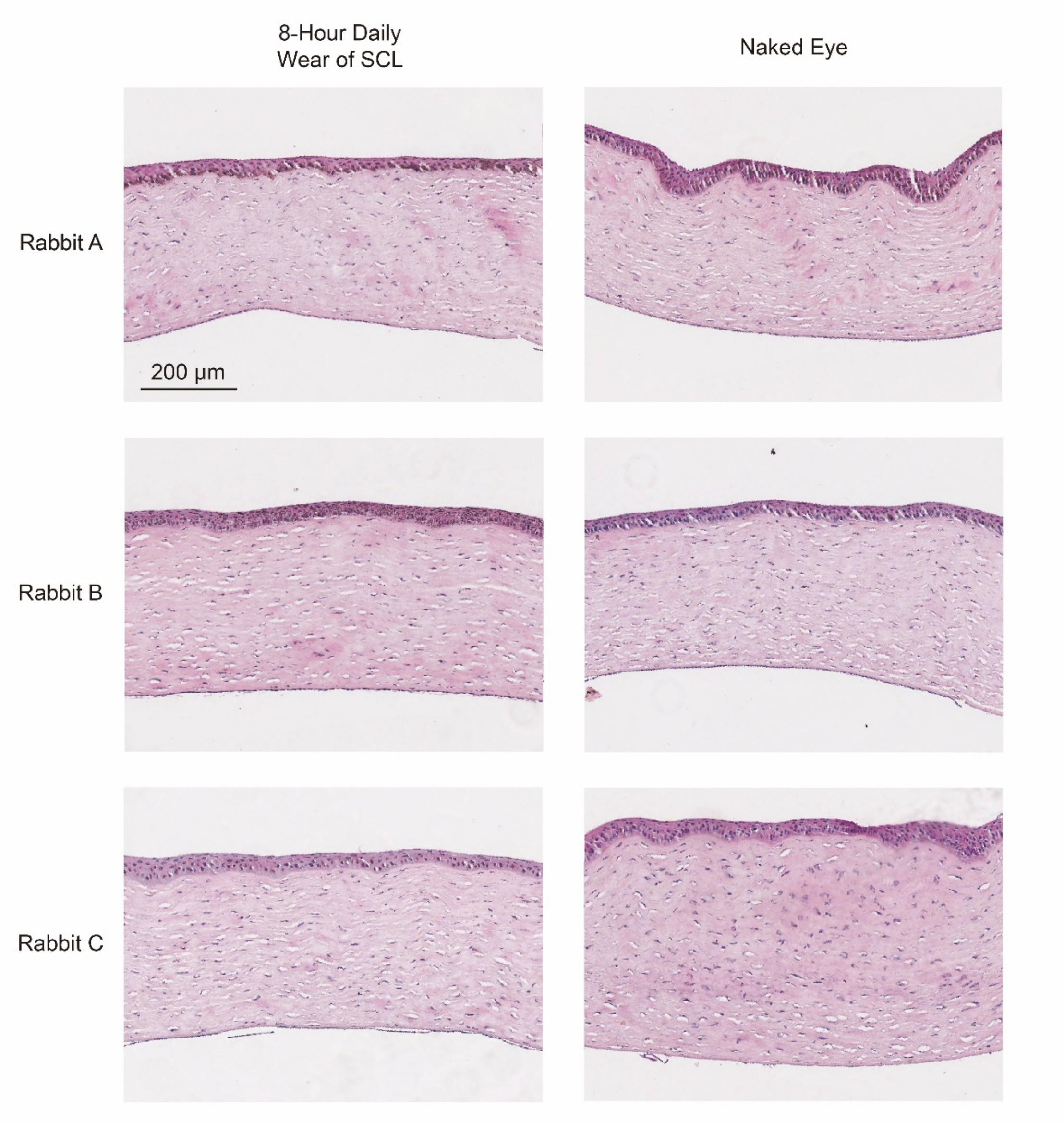

Supplementary Figure 34. Micrographs of the corneal tissue of the rabbit eyes with H&E staining after wearing the SCL for 8 hours daily over 1 week, in comparison to the naked eye.

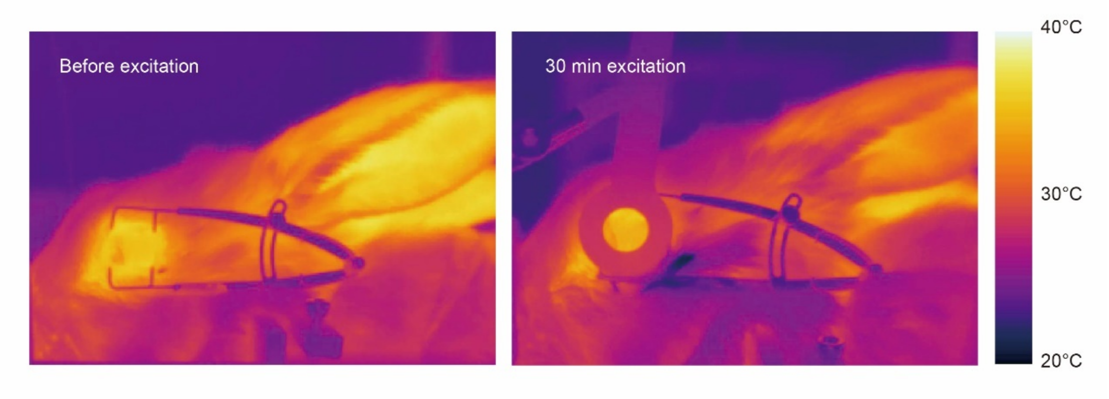

Supplementary Figure 35. IR images of the live rabbit wearing the eye track SCL before and after 30 min excitation.

Supplementary Table 1. Structural characteristic parameters of the 4 RF tags in the eye tracking SCL.

Number of Tag	Turns	Width (μm)	Gap (μm)	Inner diameter (mm)	Outer diameter (mm)
Tag-1	6	80	18	3.5	4.75
Tag-2	5	80	37	3.5	4.75
Tag-3	4	80	66	3.5	4.75
Tag-4	3.5	80	87	3.5	4.75

Supplementary Table 2. A summary of eye tracking techniques with advantages and disadvantages.

**Method**	**Mechanism**	**Components**	**Advantages**	**Disadvantages**	**Applications**
Infrared oculography (IOG)[1]	Pupil Center Corneal Reflection	Cameras and infrared lights	• Noninvasive<br>• High temporal and spatial resolution	• Interference from environmental light<br>• Individual difference<br>• High power and computations requirement	• Commercial analyzing<br>• Virtual reality
Video oculography (VOG)[2]	Image recognition	Cameras	• Noninvasive<br>• Simple hardware, inexpensive	• Same as IOG<br>• Interference from camera slippage	• Commercial analyzing<br>• Virtual reality
Electrooculography[3]	Retinal electrostatic potential	Skin-integrated electrodes	• Eye tracking with eyes closed	• Low spatial resolution<br>• Skin injury	• Medical fields
Scleral search coil[4]	Magnetic induction	Wired coil scleral lens	• Ultrahigh temporal and spatial resolution	• Wired sensor<br>• Uncomfortable<br>• Easy to slip	• Medical fields
TENG-based tracker[5]	Triboelectrification and electrostatic induction	Layered structure of dielectric layer and electrode films	• Simple structure, inexpensive<br>• Eye closing detection<br>• Noninvasive	• Limited spatial resolution	• Wearable electronics<br>• Virtual reality
Magnetic resonance-based tracker[6]	Magnetic resonance imaging	MRI scanners	• Brain activities analyzing simultaneously	• Rely on cumbersome equipment	• Medical fields
**This work**	RLC resonator	Frequency encoded contact lens	• High spatial resolution<br>• High robustness<br>• Great biocompatibility	• Complicated settings	• Virtual reality<br>• Human-machine interaction<br>• Medical fields

Supplementary Note 1:

Time-sequential eye tracking algorithm.

As shown in Figure S3, the eye tracking algorithm calculates the gazing point frame by frame and can reduce the threat of the time-varying common-mode drift by calculating the signal difference between adjacent frames. 2 preconditions (the response model and the initial fixation point) are needed to employ the algorithm and are easy to be meet. When calculating the coordinate of the fixation point at each frame, first the estimated current-frame coordinate $[x_{i,est}, y_{i,est}]$ is initialized using last-frame coordinate $[x_{i-1}, y_{i-1}]$. Tag values difference is calculated between the measured values and the ones of the response model at $[x_{i,est}, y_{i,est}]$:

$$\begin{bmatrix} \Delta Tag-1_i \\ \Delta Tag-2_i \\ \Delta Tag-3_i \\ \Delta Tag-4_i \end{bmatrix} = \begin{bmatrix} Tag-1_{mea} \\ Tag-2_{mea} \\ Tag-3_{mea} \\ Tag-4_{mea} \end{bmatrix} - \begin{bmatrix} Tag-1 \\ Tag-2 \\ Tag-3 \\ Tag-4 \end{bmatrix}_{(x_{i,est}, y_{i,est})}, \quad (1)$$

and the gradient of the model at $[x_{i,est}, y_{i,est}]$ is also calculated as:

$$\begin{bmatrix} \frac{d(Tag-1)}{dx} & \frac{d(Tag-1)}{dy} \\ \frac{d(Tag-2)}{dx} & \frac{d(Tag-2)}{dy} \\ \frac{d(Tag-3)}{dx} & \frac{d(Tag-3)}{dy} \\ \frac{d(Tag-4)}{dx} & \frac{d(Tag-4)}{dy} \end{bmatrix}_{(x_{i,est}, y_{i,est})}. \quad (2)$$

The displacement vector $[\Delta x, \Delta y]$ can be linear fitted out by the following equation:

$$\begin{bmatrix} \frac{d(Tag-1)}{dx} & \frac{d(Tag-1)}{dy} \\ \frac{d(Tag-2)}{dx} & \frac{d(Tag-2)}{dy} \\ \frac{d(Tag-3)}{dx} & \frac{d(Tag-3)}{dy} \\ \frac{d(Tag-4)}{dx} & \frac{d(Tag-4)}{dy} \end{bmatrix}_{(x_{i,est}, y_{i,est})} \cdot \begin{bmatrix} \Delta x \\ \Delta y \end{bmatrix} = \begin{bmatrix} \Delta Tag-1_i \\ \Delta Tag-2_i \\ \Delta Tag-3_i \\ \Delta Tag-4_i \end{bmatrix}. \quad (3)$$

Then the refreshed current-frame coordinate $[x_{i,ref}, y_{i,ref}]$ is calculated by:

$$\begin{bmatrix} x_{i,ref} \\ y_{i,ref} \end{bmatrix} = \begin{bmatrix} x_{i,est} \\ y_{i,est} \end{bmatrix} + \begin{bmatrix} \Delta x \\ \Delta y \end{bmatrix}. \quad (4)$$

If the difference between the measured values and the ones of the response model at $[x_{i,ref}, y_{i,ref}]$ is less than the specific threshold value, then the refreshed current-frame coordinate is regard as the result, else the estimated current-frame coordinate is covered by the refreshed one and repeating the above calculation process.

References

1. Ebisawa, Y. & Fukumoto, K. Head-Free, Remote Eye-Gaze Detection System Based on Pupil-Corneal Reflection Method with Easy Calibration Using Two Stereo-Calibrated Video Cameras. *IEEE Trans. Biomed. Eng.* **60**, 2952-2960 (2013).
2. Baek, S.J., Choi, K.A., Ma, C., Kim, Y.H. & Ko, S.J. Eyeball model-based iris center localization for visible image-based eye-gaze tracking systems. *IEEE Tran. Consum. Electron.* **59**, 415-421 (2013).
3. Homayounfar, S.Z. et al. Multimodal Smart Eyewear for Longitudinal Eye Movement Tracking. *Matter* **3**, 1275-1293 (2020).
4. Robinson, D.A. A Method of Measuring Eye Movemnent Using a Scieral Search Coil in a Magnetic Field. *IEEE Trans. Bio-Med. Electro.* **10**, 137-145 (1963).
5. Shi, Y. et al. Eye tracking and eye expression decoding based on transparent, flexible and ultra-persistent electrostatic interface. *Nat. Commun.* **14**, 3315 (2023).
6. Frey, M., Nau, M. & Doeller, C.F. Magnetic resonance-based eye tracking using deep neural networks. *Nat. Neurosci.* **24**, 1772-1779 (2021).
