# Supplementary material for: Frequency-encoded eye tracking smart contact lens for human–machine interaction
Source: Nat Commun. 2024 Apr 27;15:3588. doi: 10.1038/s41467-024-47851-y (PMC11055864; doi:10.1038/s41467-024-47851-y)
Supplement: Supplementary file 3 — Description of Additional Supplementary Files [file 41467_2024_47851_MOESM3_ESM.pdf]

<u>Description of Additional Supplementary Files</u>

Supplementary Movies

Supplementary Movie 1. Gluttonous Snake Control by SCL.

Supplementary Movie 2. Web interaction by SCL.

Supplementary Movie 3. PTZ Camera Control by SCL.

Supplementary Movie 4. Robot Vehicle Control by SCL.
