# Supplementary material for: Frequency-encoded eye tracking smart contact lens for human–machine interaction
Source: Nat Commun. 2024 Apr 27;15:3588. doi: 10.1038/s41467-024-47851-y (PMC11055864; doi:10.1038/s41467-024-47851-y)
Supplement: Supplementary file 8 — Reporting Summary [file 41467_2024_47851_MOESM8_ESM.pdf]

nature portfolio

Corresponding author(s): Fei Xu, Songtao Yuan, Yanqing Lu, and Ye Chen

Last updated by author(s): Mar 25, 2024

Reporting Summary

Nature Portfolio wishes to improve the reproducibility of the work that we publish. This form provides structure for consistency and transparency in reporting. For further information on Nature Portfolio policies, see our Editorial Policies and the Editorial Policy Checklist.

Statistics

For all statistical analyses, confirm that the following items are present in the figure legend, table legend, main text, or Methods section.

n/a	Confirmed	
☐	☒	The exact sample size (*n*) for each experimental group/condition, given as a discrete number and unit of measurement
☐	☒	A statement on whether measurements were taken from distinct samples or whether the same sample was measured repeatedly
☐	☒	The statistical test(s) used AND whether they are one- or two-sided<br>*Only common tests should be described solely by name; describe more complex techniques in the Methods section.*
☒	☐	A description of all covariates tested
☒	☐	A description of any assumptions or corrections, such as tests of normality and adjustment for multiple comparisons
☐	☒	A full description of the statistical parameters including central tendency (e.g. means) or other basic estimates (e.g. regression coefficient) AND variation (e.g. standard deviation) or associated estimates of uncertainty (e.g. confidence intervals)
☐	☒	For null hypothesis testing, the test statistic (e.g. *F*, *t*, *r*) with confidence intervals, effect sizes, degrees of freedom and *P* value noted<br>*Give P values as exact values whenever suitable.*
☒	☐	For Bayesian analysis, information on the choice of priors and Markov chain Monte Carlo settings
☒	☐	For hierarchical and complex designs, identification of the appropriate level for tests and full reporting of outcomes
☒	☐	Estimates of effect sizes (e.g. Cohen's *d*, Pearson's *r*), indicating how they were calculated

Our web collection on statistics for biologists contains articles on many of the points above.

Software and code

Policy information about availability of computer code

Data collection	The experiments of calligraphy and painting by eye tracking, eye-machine interaction by eye command input, and in vivo eye-machine interaction were realized based on LabVIEW (2019).
Data analysis	Eye-drawing 'NJU' letters and 'snake' pattern were realized based on MATLAB R2020b (MathWorks).<br>Origin Pro (Origin Lab)

For manuscripts utilizing custom algorithms or software that are central to the research but not yet described in published literature, software must be made available to editors and reviewers. We strongly encourage code deposition in a community repository (e.g. GitHub). See the Nature Portfolio guidelines for submitting code & software for further information.

Data

Policy information about availability of data

All manuscripts must include a data availability statement. This statement should provide the following information, where applicable:

- Accession codes, unique identifiers, or web links for publicly available datasets
- A description of any restrictions on data availability
- For clinical datasets or third party data, please ensure that the statement adheres to our policy

The authors declare that all data supporting the results in this study are present in the paper and the data sources are uploaded in Supplementary Information with

this paper. Any additional requests for information can be directed to, and will be fulfilled by, the corresponding authors. Source data are provided with this paper. The codes supporting this study's findings are available from https://github.com/yiyinju/Time-sequence-eye-tracking-algorithm

Research involving human participants, their data, or biological material

Policy information about studies with human participants or human data. See also policy information about sex, gender (identity/presentation), and sexual orientation and race, ethnicity and racism.

Reporting on sex and gender	N/A
Reporting on race, ethnicity, or other socially relevant groupings	N/A
Population characteristics	N/A
Recruitment	N/A
Ethics oversight	N/A

Note that full information on the approval of the study protocol must also be provided in the manuscript.

Field-specific reporting

Please select the one below that is the best fit for your research. If you are not sure, read the appropriate sections before making your selection.

☒ Life sciences ☐ Behavioural & social sciences ☐ Ecological, evolutionary & environmental sciences

For a reference copy of the document with all sections, see nature.com/documents/nr-reporting-summary-flat.pdf

Life sciences study design

All studies must disclose on these points even when the disclosure is negative.

Sample size	Sample size was determined based on reference. In long-term cytotoxicity test of SCL, 6 wells of HCE-T were counted. In long-term in vivo eye irritation tests, 3 New Zealand white rabbits were employed to wear the SCLs.
Data exclusions	No data were excluded.
Replication	Replication of the eye tracking SCL was successful. Eye-drawing NJU letters were realized using 4 different samples. Repeatability and robustness of the SCL were conprehensively assessed by multiple tests in this paper. These tests included anti-interference tests for ambient light and common radio frequency sources and accuracy tests for eye tracking with varying corneal curvatures and reading distances. And replication of cytotoxicity test and eye irritation tests of SCL was successful.
Randomization	In biocompatibility tests using in vivo rabbits, the left eye worn the eye-tracking SCL and the right eye naked or worn a commercial contact lens as a comparison for each rabbit. The eye picked for wearing the SCL was identical and would not affect the objectivity of the results, because there was no difference whether the SCL was worn with the left eye or the right eye and both eyes experienced the same feeding conditions.
Blinding	Blinding was not relevant because no variant would be changed by blinding.

Reporting for specific materials, systems and methods

We require information from authors about some types of materials, experimental systems and methods used in many studies. Here, indicate whether each material, system or method listed is relevant to your study. If you are not sure if a list item applies to your research, read the appropriate section before selecting a response.

Materials & experimental systems

n/a	Involved in the study
☒	☐ Antibodies
☐	☒ Eukaryotic cell lines
☒	☐ Palaeontology and archaeology
☐	☒ Animals and other organisms
☒	☐ Clinical data
☒	☐ Dual use research of concern
☒	☐ Plants

Methods

n/a	Involved in the study
☒	☐ ChIP-seq
☒	☐ Flow cytometry
☒	☐ MRI-based neuroimaging

Eukaryotic cell lines

Policy information about cell lines and Sex and Gender in Research

Cell line source(s)	HCE-T (Cellosaurus; CVCL_1272) were obtained from Shanghai Zhong Qiao Xin Zhou Biotechnology Co.,Ltd. The sex of HCE-T was female and the cells were derived from human participants.
Authentication	The authentication of cell lines was performed using short tandem repeat (STR) profiling. The results matched the markers of HCE-T in Cellosaurus database.
Mycoplasma contamination	All cell lines tested negative for mycoplasma.
Commonly misidentified lines (See ICLAC register)	No commonly misidentified cell lines were used.

Animals and other research organisms

Policy information about studies involving animals; ARRIVE guidelines recommended for reporting animal research, and Sex and Gender in Research

Laboratory animals	New Zealand white rabbits (3 months old)
Wild animals	No wild animal was involved.
Reporting on sex	5 Female New Zealand white rabbits were employed. The study's findings were independent of sex.
Field-collected samples	The study did not include data collected from field.
Ethics oversight	All animal experiments were performed according to the National Institute of Health Guidelines under the protocols approved by the ethics committee at the Nanjing Medical University (SYXK(Su)2021-0023).

Note that full information on the approval of the study protocol must also be provided in the manuscript.

Plants

Seed stocks	N/A
Novel plant genotypes	N/A
Authentication	N/A
